# A bacterial symbiont in the gill of the marine scallop *Argopecten irradians irradians* metabolizes dimethylsulfoniopropionate

**DOI:** 10.1002/mlf2.12072

**Published:** 2023-06-26

**Authors:** Yi Shu, Yongming Wang, Zhongcheng Wei, Ning Gao, Shuyan Wang, Chun‐Yang Li, Qiang Xing, Xiaoli Hu, Xiao‐Hua Zhang, Yu‐Zhong Zhang, Weipeng Zhang, Zhenmin Bao, Wei Ding

**Affiliations:** ^1^ MOE Key Laboratory of Marine Genetics and Breeding Ocean University of China Qingdao China; ^2^ Laboratory of Tropical Marine Germplasm Resources and Breeding Engineering, Sanya Oceanographic Institution Ocean University of China Sanya China; ^3^ College of Marine Life Sciences Ocean University of China Qingdao China; ^4^ Institute of Evolution & Marine Biodiversity Ocean University of China Qingdao China

**Keywords:** *Alphaproteobacteria*, *dddP*, DMSP lyases, scallop, symbiont

## Abstract

Microbial lysis of dimethylsulfoniopropionate (DMSP) is a key step in marine organic sulfur cycling and has been recently demonstrated to play an important role in mediating interactions between bacteria, algae, and zooplankton. To date, microbes that have been found to lyse DMSP are largely confined to free‐living and surface‐attached bacteria. In this study, we report for the first time that a symbiont (termed “*Rhodobiaceae* bacterium HWgs001”) in the gill of the marine scallop *Argopecten irradians irradians* can lyse and metabolize DMSP. Analysis of 16S rRNA gene sequences suggested that HWgs001 accounted for up to 93% of the gill microbiota. Microscopic observations suggested that HWgs001 lived within the gill tissue. Unlike symbionts of other bivalves, HWgs001 belongs to *Alphaproteobacteria* rather than *Gammaproteobacteria*, and no genes for carbon fixation were identified in its small genome. Moreover, HWgs001 was found to possess a *dddP* gene, responsible for the lysis of DMSP to acrylate. The enzymatic activity of *dddP* was confirmed using the heterologous expression, and in situ transcription of the gene in scallop gill tissues was demonstrated using reverse‐transcription PCR. Together, these results revealed a taxonomically and functionally unique symbiont, which represents the first‐documented DMSP‐metabolizing symbiont likely to play significant roles in coastal marine ecosystems.

## Impact statement

This is the first description of a taxonomically and functionally unique dimethylsulfoniopropionate (DMSP)‐metabolizing symbiont that is likely to play significant roles in coastal marine ecosystems. This bacterium lives in the gill of the marine scallop *Argopecten irradians irradians* and is likely to use DMSP as its major carbon source. The findings expand the current understanding of the lifestyles of animal symbionts and open a new perspective for the study of marine sulfur cycling.

## INTRODUCTION

Dimethylsulfoniopropionate (DMSP) is important in the sulfur cycle in the ocean and is the main precursor of dimethylsulfide (DMS), which contributes to global climate regulation by influencing cloudiness^1^, ^2^, ^3^, ^4^. As the most abundant organic sulfur compound in marine environments, DMSP is produced mainly by phytoplankton in amounts as high as several hundred million tons annually^1^, ^5^, ^6^, ^7^. Meanwhile, DMSP is degraded by marine bacteria through the demethylation pathway^8^, cleavage pathways^9^, the oxidation pathway^5^, and a recently‐reported pathway where it is cleaved by the addition of CoA^10^. At the molecular level, DddP is one of the most widespread bacterial DMSP lyases in the ocean^11^, ^12^, ^13^.

Marine bacteria have three major lifestyles, namely, free‐living (bacterioplankton), biofilm association (sessile bacteria), and symbiosis (plant or animal symbionts). Most of the known DMSP‐degrading bacteria are bacterioplankton while a few are surface‐associated bacteria. For example, free‐living members of *Pelagibacterales* (previously known as SAR11), SAR116, and *Roseobacter* were demonstrated to degrade DMSP by the cleavage pathway in marine environments, including coastal waters, the deep sea, and polar regions^7^, ^14^, ^15^. In addition, several biofilm‐associated bacteria, such as *Spongiobacter*, *Pseudomonas*, *Roseobacter*, and *Vibrio* spp., isolated from coral surfaces are also able to metabolize DMSP^16^. However, no symbiotic bacteria, especially typical symbionts living in the tissue of marine animals, have been reported to degrade DMSP, despite the well‐recognized fact that symbiosis with marine animals is a common and ecologically important lifestyle for marine bacteria.

Bivalves living in coastal marine environments have both economic and ecological importance. In China, the bay scallop (*Agropectans irradians irradians*, *Pectinidae*, *Mollusca*) is one of the most important shellfish species. In 2007, the production of the bay scallop reached about 600,000 tons due to both its large adductor muscle and rapid growth rate^17^. Moreover, these bivalves feed mainly on algae and are thus considered important reservoirs of DMSP^18^, ^19^. The concentrations of DMSP in scallop gills can reach 21 µg/g^20^. In addition, the symbiosis between chemosynthetic bacteria and bivalves (e.g., mussels, clams, and oysters) is ubiquitous; hundreds of bivalve species have been described, many belonging to the family *Mytilidae* ^21^, ^22^, ^23^, ^24^, ^25^, which rely on bacterial symbionts for carbon fixation, energy production, vitamin biosynthesis, or detoxification. However, surprisingly, the symbionts living in scallops remain largely unknown.

In this study, we explored the community structure of microbiota in the gill tissues of coastal scallops and found a symbiotic bacterium that dominates the gill has the potential to degrade DMSP. A combination of 16S rRNA gene amplicon sequencing, fluorescence in situ hybridization (FISH), genome binning, heterogeneous expression, and enzymatic assays was used to elucidate the relevant molecular mechanisms and ecological significance of this symbiont.

## RESULTS

### An intracellular symbiont dominated the gill microbiota of *A. i. irradians*

Nearly full‐length 16S rRNA genes (the primers are shown in Table S1, and the sequencing information are shown in Table S2) were used to profile the taxonomy of the gill microbiota from seven *A. i. irradians* individuals. Three adjacent seawater samples were also analyzed as references. The smooth rarefaction curves toward the end of sampling indicated that saturation of microbial richness had been achieved (Figure S1). In each of the seven scallop gill samples, less than 220 operational taxonomic units (OTUs) were detected, compared to more than 400 OTUs identified in each of the adjacent seawater samples (Figure S1), suggesting that the microbial diversity in the gill microbiota was significantly lower than that in the seawater. In addition, lower average values of both Shannon and Chao_1 diversity were observed in the gill samples compared with the seawater samples (Figure S2). There was a significant difference between the alpha diversity of the gill and that of the seawater, as confirmed by Student's *t*‐test (*p* < 0.001). The dissimilarity between the gill microbiota and seawater was visualized by principal coordinate analysis (PCoA) based on the composition of the OTUs. A clear difference between the two groups was revealed as the PCoA1 explained 97.38% of the difference (Figure S3).

After the classification of the 16S rRNA genes (8000 sequences per sample) at the phylum level, all samples were classified into 30 taxa (*Proteobacteria* were classified up to the class level) (Figure S4). Notably, as the dominant taxa, *Alphaproteobacteria* accounted for 61%–93% of the gill microbiota, whereas *Gammaproteobacteria* were over‐represented in the seawater microbial communities, followed by *Alphaproteobacteria* and *Cyanobacteria* (Figure S4). At the genus level, an unclassified member of the family *Rhodobiaceae* (*Rhodobiaceae*_Group; *Rhizobiales*; *Alphaproteobacteria*) accounted for more than 52% of the gill microbiota, whereas it was undetectable in seawater (Figure S5). The *Rhodobiaceae*_Group was further classified to the OTU level, and it was represented by one dominant OTU (OTU_322591; Figure S6). Thus, we speculated that this OTU might correspond to a bacterium living in the gill tissue, considering that many previously documented intracellular microbiota are known to be dominated by only one species^26^.

**Figure 1:**
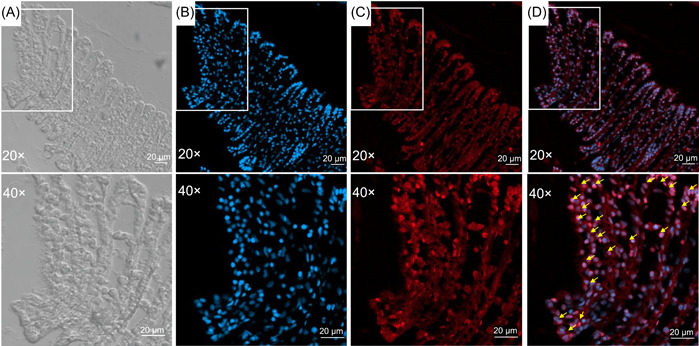
Fluorescence in situ hybridization (FISH) images of the gill filament at 20× and 40× magnification. (A) Phase‐difference micrograph of the gill filament. (B) Gill cells stained with 4′,6‐diamidino‐2‐phenylindole (DAPI) (blue). (C) FISH image using an oligo‐DNA probe specifically targeting the 16S rRNA of the OTU_322591 (red); (D) The merged image of the OTU_322591 signals (red) and the DAPI‐stained gill cells (blue). The white boxes represent accumulated bacterial cells in the gill tissues which are shown by yellow arrows. Scale bars, 20 µm.

As expected, fluorescent in situ hybridization (FISH) experiments confirmed the presence of this bacterium in the gill tissue (Figure 1). Specifically, we observed aggregating bacteria in the gill tissue, indicated as positive signals from the specific probes targeting OTU_322591 (Figure 1). Moreover, it was found that the OTU_322591 signals (red) and the 4′,6‐diamidino‐2‐phenylindole (DAPI)‐stained gill cells (blue) were consistently overlapped (Figure 1D), suggesting that the symbiont was likely to be intracellular. The presence of intracellular endosymbionts tightly packed inside bacteriocytes was confirmed by transmission electron microscopy (TEM) of the gill tissues (Figure S7). These results thus confirmed that the scallop gill is a likely holobiont with two active parties, namely, the host and the symbiont in the gill tissue.

### Overall genomic features and phylogeny of the gill symbiont

**Figure 2:**
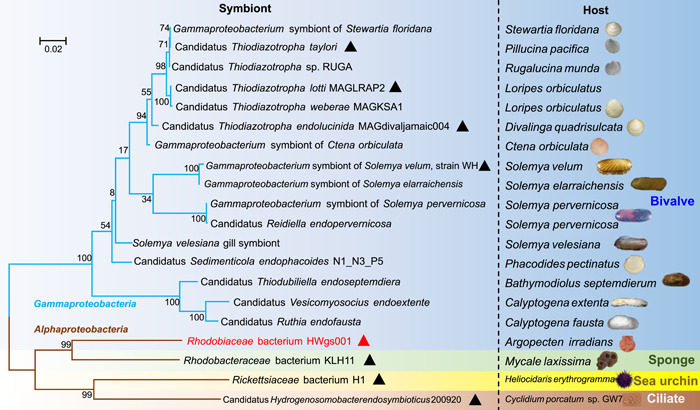
Phylogenetic relationships between the scallop symbiont HWgs001 and symbiont bacteria of other bivalves. Three alphaproteobacterial symbionts from the sponge *Mycale laxissima*, the sea urchin *Heliocidaris*, and the ciliate *Cyclidium porcatum* are also included in the analysis. The tree was built based on 16S rRNA gene sequences in a maximum‐likelihood mode. Bootstrap values were generated through 1000 replicated calculations and are shown on the branches. Triangles indicate symbionts whose sequences were used for subsequent functional genomic comparisons. The respective hosts are shown in the rightmost column.

Hereafter, the symbiont was designated as *Rhodobiaceae* bacterium HWgs001. To obtain genomic information on this bacterium, metagenomic sequencing was performed on the whole microbial community of the gill, resulting in 61.99 Gb of reads. These DNA reads were further assembled into 1,264,685 contigs, and after genome binning, the symbiont genome with 92.33% completeness and 0% contamination was obtained. Information on the metagenomic data is shown in Table S3, and the genome information is shown in Table S4. The genome size was rather small (1.71 Mb), with 36.03% GC content, 1564 open reading frames (ORFs), a single operon of three rRNA genes, and 30 tRNA genes (Table S4). We next sought to reveal its phylogenetic relationship with symbionts of other marine animals. A total of 19 bacterial symbionts documented in previous studies served as references to construct a tree with HWgs001 (Figure 2). These reference symbionts were from ciliate, sea urchins, sponges, and a variety of bivalves (Figure 2). Notably, HWgs001 was located on a branch separate from the symbionts of other bivalves, which were affiliated to the *Gammaproteobacteria* class (Figure 2). In contrast, HWgs001 was relatively closer to the sponge symbiont *Rhodobacteraceae* bacterium KLH11 (Figure 2), with a 16S rRNA gene identity of 84% (Figure S8). Moreover, searching against the National Center for Biotechnology Information (NCBI) databases revealed sequence identities below 89% (Figure S9). Together, these findings suggested that HWgs001 is probably a novel symbiont with a streamlined genome and a unique origin.

### Metabolic pathway reconstruction

**Figure 3:**
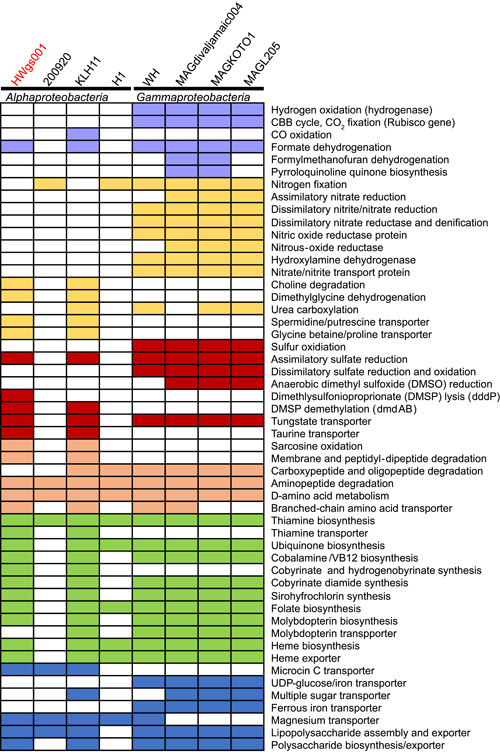
Functional genomic comparison between the scallop symbiont HWgs001 and seven other symbionts. 200920, Candidatus *Hydrogenosomobacter endosymbioticus* 200920 from the ciliate *Scuticociliate*; H1, *Rickettsiaceae* bacterium H1 from the sea urchin *Heliocidaris*; KLH11, *Rhodobacteraceae* bacterium KLH11 from the sponge *Mycale laxissima*; MAGL205, Candidatus *Thiodiazotropha taylori* MAGL205 from the bivalve *Loripes orbiculatus*; MAGdivaljamaic004, Candidatus *Thiodiazotropha endolucinida* MAGdivaljamaic004 from the bivalve *Loripes orbiculatus*; MAGKOTO1, Candidatus *Thiodiazotropha lotti* MAGKOTO1 from the bivalve *Loripes orbiculatus*; WH, *Gammaproteobacterium* symbiont of the bivalve *Solemya velum*, strain WH; the phylogenetic relationships between these bacteria and their hosts are shown in Figure 2. The colored box indicates the existence of the corresponding pathway.

We next performed Kyoto Encyclopedia of Genes and Genomes (KEGG) annotation and functional analyses on ORFs derived from the HWgs001 genome and compared the major functions with seven other symbionts. Overall, the eight genomes shared 258 KEGGs (Figure S10A), including pathways related to DNA repair, ribosome assembly, secondary metabolite biosynthesis, lipid biosynthesis, peptide degradation, secretion, and transport (Figure S10B). Individually, these bacteria had 25–665 specific KEGGs (Figure S10A), suggesting the presence of significant functional variation. Further comparison between HWgs001 and other animal symbionts revealed specific functions associated with the former, including hydrogen oxidation, carbon fixation, nitrogen, sulfur, and peptide metabolism, vitamin and cofactor biosynthesis, as well as virulence and biofilm formation (Figure 3). Hydrogenase, which catalyzes the reversible oxidation of molecular hydrogen, was absent in HWgs001, whereas over 10 copies of this gene were identified in WH, MAGdivaljamaic004, MAGKOTO1, and MAGL205 (Figure 3). In terms of carbon fixation, genes encoding the large and small subunits of the ribulose‐bisphosphate carboxylase were present in WH, MAGdivaljamaic004, MAGKOTO1, and MAGL205, and a gene encoding carbon‐monoxide dehydrogenase (CO oxidation) was identified in KLH11, whereas no genes for carbon dioxide or carbon monoxide fixation were identified in HWgs001 (Figure 3). In terms of nitrogen metabolism, assimilatory metabolism‐related genes such as those involved in choline degradation and dimethylglycine degradation were only found in HWgs001 and KLH11, whereas genes for dissimilatory nitrate and nitrite reduction were found in WH, MAGdivaljamaic004, MAGKOTO1, and MAGL205, and nitrogen fixation proteins were found in all eight genomes except HWgs001 and KLH11 (Figure 3). In terms of sulfur metabolism, genes associated with sulfur oxidation and dissimilatory sulfate reduction and oxidation were present in WH, MAGdivaljamaic004, MAGKOTO1, and MAGL205 but were absent in the other four strains. However, the DMSP lysis gene (*dddP*) was specifically detected in HWgs001 while DMSP demethylation genes (*dmdAB*) were detected in HWgs001 and KLH11 (Figure 3). Genes for amino acid metabolism were detected in all eight strains, and especially, branched‐chain amino acid transporters were present in the genomes of HWgs001, KLH11, WH, and MAGdivaljamaic004 (Figure 3). Genes associated with thiamine biosynthesis were detected in all eight strains, whereas genes belonging to vitamin B12 and cofactor biosynthesis were present in the genomes of HWgs001, KLH11, WH, MAGdivaljamaic004, MAGKOTO1, and MAGL205, but most of them were absent in the genomes of 200920 and H1 (Figure 3). In addition, genes for virulence and biofilm formation were identified, for example, the microcin C transport system was identified in HWgs001, 200920, and KLH11 (Figure 3).

**Figure 4:**
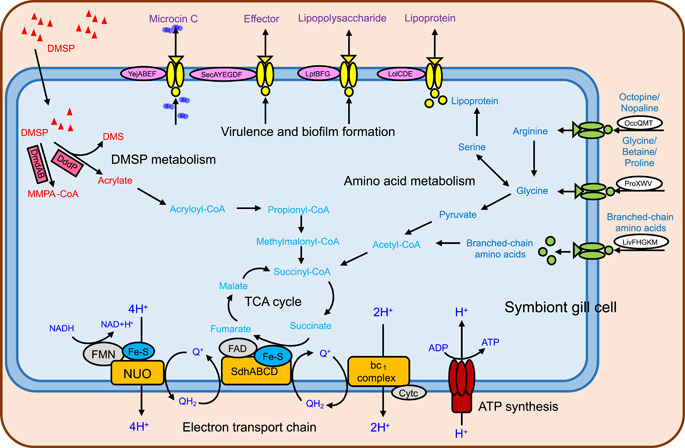
A schematic model showing the major carbon metabolic pathways in HWgs001. Dimethylsulfoniopropionate (DMSP) is cleaved to produce dimethylsulfide (DMS) and acrylate. Acrylate is transformed into acryloyl‐CoA, and finally, succinyl‐CoA enters the TCA cycle. FAD, flavin adenine dinucleotide; FMN, flavin mononucleotide; NUO, NADH dehydrogenase complex.

Based on the KEGG annotation, we reconstructed metabolic pathways in HWgs001 (Figure 4). The absence of genes for inorganic carbon fixation suggested that HWgs001 is completely reliant on heterotrophic carbon metabolism. Notably, a complete pathway of DMSP metabolism was reconstructed, where DMSP was cleaved into DMS and acrylate, after which the acrylate was transformed into acryloyl‐CoA, propionyl‐CoA, methylmalonyl‐CoA, and finally succinyl‐CoA, which then entered the tricarboxylic acid cycle (TCA) cycle for the production of GTP and NADH (Figure 4). The acquired branched‐chain amino acids (valine, leucine, and isoleucine), glycine, and arginine could be transformed into pyruvate and enter the TCA cycle (Figure 4). In terms of cell membrane‐supported energy production (oxidative phosphorylation), HWgs001 used the proton‐pumping NADH dehydrogenase complex (NUO or complex I), the SdhABCD complex (complex II), the cytochrome bc1 complex, and ATPase for electron transport and ATP synthesis (Figure 4). The virulence and biofilm formation‐related pathways, including the secretion of microcin C, Sec effector, lipopolysaccharide, and lipoprotein, were also plotted, as shown in Figure 4. In addition, based on the holobiont concept, the host and its symbiont may have genes that are functionally related^27^. We thus annotated the ORFs derived from the scallop genome^28^, and no genes for DMSP degradation could be identified, implying that the scallop itself is not able to metabolize DMSP.

### Enzymatic activity and in situ gene expression evidence for DMSP lysis

**Figure 5:**
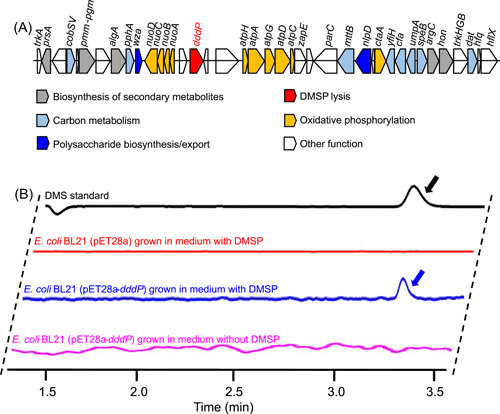
Distribution pattern of the *dddP* gene in the genome of HWgs001 and its enzyme activity. (A) The *dddP* gene is adjacent to genes involved in energy metabolism. The gene cluster was drawn using a Python script. (B) The *dddP* gene was expressed in *Escherichia coli* BL21, and the dimethylsulfoniopropionate (DMSP) lysis activity was detected by measuring the dimethylsulfide (DMS) production using gas chromatography. The peaks indicated by arrows correspond to DMS.

To gain more insight into DMSP metabolism, we conducted reverse‐transcription PCR (RT‐PCR), metatranscriptomic analysis, and enzyme assays to examine the in situ expression and in vitro function of genes associated with DMSP metabolism. RT‐PCR (the primers are shown in Table S1) and metatranscriptomic analysis were performed on scallop gill tissues collected in the coastal water of Qingdao and immediately fixed for RNA extraction. RT‐PCR indicated that *dddP*, *dmdA*, and *dmdB* were expressed in the gill tissues (Figure S11). All three genes showed high levels of expression (transcripts per million (TPM) >100), with the TPM of *dmdA* or *dmdB* being higher than that of *dddP* (Figure S12), supporting the role of HWgs001 in DMSP metabolism and transformation. Before investigating the in vitro functions of the DMSP lyase, genes adjacent to *dddP* in the HWgs001 genome were visualized to eliminate the possibility that the presence of this gene was not caused by contig contamination. It was found that *dddP* was distributed in a long contig (more than 11 kb) and adjacent to operons related to oxidative phosphorylation, namely, the *nuo* and the *atp* operons (Figure 5A). Online search against the NCBI‐Nr database revealed that genes within the *nuo* and the *atp* operons are affiliated to *Rhodobiaceae*, suggesting that the presence of the *dddP* gene was not the result of contig contamination. A phylogenetic analysis of all *dddP* genes from previously documented DMSP consumers was then performed. On the phylogenetic tree, the DddP protein from HWgs001 was located on an independent branch with the reference DddP proteins (Figure S13). The most closely related DddP (identity = 55%) was DddP2 (G5CZE7) from *Oceanimonas doudoroffii* (Figure S14), which has been demonstrated to be a *Gammaproteobacterium* with multi‐DMSP lyases^29^, whereas DddP1 (G5CZD9) from *O. doudoroffi* was located on a separate branch (Figure S13). In addition, the DddP protein sequence from HWgs001 aligned with the sequences of other DddP proteins, and the alignment showed the presence of conserved active sites (namely D263, D265, D275, H339, E385, and E390) that were consistent with those identified in *Roseovarius nubinhibens* DddP (A3SK19)^11^ (Figure S14). We then cloned and expressed *dddP* in *Escherichia coli* BL21, and the sodium dodecyl sulfate‐polyacrylamide gel electrophoresis (SDS‐PAGE) result indicated that DddP was a protein with a molecular weight of approximately 55 kDa (Figure S15), which was consistent with the theoretical prediction. The DMSP cleavage activity of the HWgs001 DddP protein was then studied, which showed abundant DMS exclusively in the recombinant bacterium grown with DMSP (Figure 5B), suggesting DMSP cleavage activity by the DddP protein.

## DISCUSSION

This study focused on the gill microbiota of the scallop *A. i. irradians* living in coastal waters. The results of 16S rRNA gene sequencing and FISH identified a symbiont (designated HWgs001) with a unique taxonomic affiliation. We investigated and identified potential metabolic interactions between HWgs001 and the host through genomic analyses. Furthermore, the biochemical experiments demonstrated the ability of HWgs001 to lyse DMSP, an ecologically important organic sulfur compound.

We propose that HWgs001 represents a novel symbiont of marine bivalves with a unique origin. First, the results of the taxonomic analysis, as well as the FISH, indicate that HWgs001 is indeed a symbiont. HWgs001 is the most abundant (>90%) OTU in the gill microbiota, while it is undetectable in adjacent seawater, suggesting its stable association with the host. Similar situations have been observed in many previous studies. For example, the gill microbiota of *Lucinidae* clams is dominated by one *Sedimenticola* (a genus under *Gammaproteobacteria*) species, with an approximate relative abundance of 84% in the community^26^. The small genome of HWgs001 is also consistent with symbiotic characteristics. Second, unlike most of the previously documented gammaproteobacterial bivalve symbionts, this bacterium is taxonomically affiliated to *Alphaproteobacteria* and occupies a unique position in the phylogenic tree. Marine bivalve symbionts living in the surface waters of deep‐sea environments are typically affiliated to *Gammaproteobacteria* ^30^, ^31^, ^32^ and, in some cases, affiliated to *Epsilonproteobacteria* ^32^, ^33^, ^34^ but rarely *Alphaproteobacteria*. In contrast, the phylogenetic analysis suggests a close evolutionary relationship between HWgs001 and an alphaproteobacterial symbiont of the marine sponge, implying that HWgs001 may have an origin distinct from those of other bivalve symbionts.

HWgs001 is likely to interact with the host in a previously unknown manner. While symbionts are not essential for host survival, they can increase the fitness of the host in specific environmental niches^35^. In the deep‐sea environment where hydrogen sulfide gas is abundant, symbionts of bivalves often possess multiple oxidation pathways associated with the reduction of sulfur compounds^32^, and the generated energy can be used to fix carbon dioxide. For example, the lucinid symbionts Candidatus *Thiodiazotropha taylori*, Candidatus *Thiodiazotropha endolucinida*, and Candidatus *Thiodiazotropha lotti* rely on multiple pathways to oxidize reduced sulfur compounds to sulfates through a polysulfur or elemental sulfur intermediate, and the energy produced is used to power inorganic carbon fixation through the Calvin–Benson–Bassham cycle^26^. In addition, lucinid symbionts were recently found to be capable of fixing inorganic nitrogen from the atmosphere^36^, ^37^. In contrast, there are relatively rich sources of organic carbon in coastal marine environments, and the DMSP concentration is higher than that in the deep sea. Thus, it would be reasonable for bivalves to recruit a symbiont not for the use of inorganic carbon or sulfur compounds but one that can metabolize DMSP. Consistently, the genomic analyses support the hypothesis that HWgs001 can lyse DMSP, and the product, acrylate, is likely to enter central carbon metabolism pathways after its transformation to acetyl‐CoA. Moreover, the similar locations of the *dddP* gene and several energy production‐related genes, as well as the absence of pathways for metabolizing complex organic material such as polysaccharides or lipids, suggests that DMSP may represent one of the major carbon sources for heterotrophic growth. This is not the first report of heterotrophic symbionts, which have been previously reported in the ciliate *Cyclidium porcatum* living in coastal sediments around the world^38^. Nevertheless, to date, no heterotrophic symbionts have been reported to metabolize DMSP, and thus our results highlight the high diversity of metabolic interactions between bacteria and their animal hosts.

The findings in the current study also shed new light on the *dddP* gene and DMSP degradation. Bacterial *dddP* genes have been suggested to be widely distributed in marine environments and are especially more common in polar deep waters^15^, ^39^. However, because previous studies have been confined to bacterioplankton, the observed presence of *dddP* genes in coastal scallops implies that their abundance and contribution to the DMSP cycle have been underestimated. Furthermore, the *dddP2* gene in *O. doudoroffii* was suggested to be phylogenetically distinct from most of the known *dddP* genes, showing considerably greater efficiency than *dddP1* in *O. doudoroffii* ^29^. Thus, the combination of our experimental evidence that HWgs001 *dddP* can efficiently degrade DMSP and the close relationship between HWgs001 *dddP* and *dddP2* in *O. doudoroffii* highlights the important role of HWgs001 *dddP* in the degradation of marine DMSP.

Together, this study represents the first description of a taxonomically and functionally unique DMSP‐metabolizing symbiont that is likely to play significant roles in coastal marine ecosystems. Considering its symbiotic features and the fact that many symbionts show vertical inheritance, it is likely that the coexistence between this bacterium and its host, the scallop, has a long evolutionary history. The occurrence of such a symbiosis might be driven by the utilization of DMSP, in line with the concept that host–microbe coadaptations are potentially driven by environmental factors^40^. Additionally, we propose that *dddP*‐mediated DMSP transformation is likely to be more common and important than previously thought. These findings will help in expanding the current understanding of the lifestyle of animal symbionts and open a new perspective for the study of marine sulfur cycling.

## MATERIALS AND METHODS

### Sample collection and DNA extraction

The samples used in this study were prepared from 1‐year‐old scallops (*A. i. irradians*) which were obtained from artificial scallop‐rearing substrates deployed in Dingjiazui Bay, Qingdao, China (35°56′41.50″ N, 120°23′14.63″ E). The encrusted organisms were removed from the shells and the scallops were transferred to the laboratory where they were kept alive in a breeding system with filtered seawater. The gill of the scallop was dissected under aseptic conditions, washed using sterile 4× phosphate‐buffered saline (PBS, pH 7.4; Solarbio), and frozen in liquid nitrogen. All the tissues were stored at −80°C until DNA and RNA extraction. The total microbial DNA in the gill tissues was extracted according to the protocol described in the QIAamp DNA microbiome kit (Qiagen) and stored at −80°C. In addition, seawater was collected from three different locations in Dingjiazui Bay^41^. After transfer to the laboratory, the seawater samples (5 l from each location) were filtered with 0.22‐µm filter membranes, which were then cut, crushed, and placed in 50 ml centrifuge tubes filled with artificial seawater and incubated with shaking for 15 min. The bacterial cells were pelleted by centrifugation at 6000 rpm and used for DNA extraction.

### Amplicon sequencing and 16S rRNA gene analysis

To explore the composition and diversity of the microbiomes in scallop gills and adjacent seawater, amplification of long reads of the 16S rRNA genes from the microbial communities in the gill tissue and surrounding seawater was performed using barcoded primers of 27F/1492R (Table S1)^41^, ^42^. The PCR reaction mixtures contained 25 μl Prime Star Max premix (R045; Takara), 2.5 μl F/R primers, and 1 μl DNA template, made up to a final volume of 50 µl with distilled H_2_O. The PCR reaction was 95°C for 5 min, followed by 30 cycles of amplification (95°C for 30 s, 57°C for 30 s, and 72°C for 2 min), and 72°C for 5 min for the final extension. The PCR products were analyzed by 2% agarose gel electrophoresis and the target bands were recovered using the QIAquick PCR & Gel Cleanup Kit (Qiagen), following the manufacturer's instructions. The DNA fragments were purified using AMPure PB magnetic beads to construct the SMRT Bell library, which was quantified by Qubit concentration. Seven libraries were constructed for the gill and three for the surrounding seawater samples. The inserted fragments were detected on an Agilent 2100 Bioanalyzer (Agilent) and sequenced on the PacBio RS II platform at Novogene Bioinformatics Technology Co., Ltd., generating more than 15,000 reads per sample.

The sequence information is shown in Table S2. The 16S rRNA gene reads were retrieved and split based on barcodes and saved in bam format. For sequence correction, circular consensus sequencing (CCS) mode was used, with CCS = 3, minimum accuracy = 0.99, and a length cutoff of 1302 bp. Clean reads were generated by the removal of primers and adapters using Cutadapt software^43^ and chimeras were filtered using Vsearch (v2.13.3)^44^. All the clean reads were merged and clustered at 97% similarity using Usearch (v8.0)^45^ to generate OTUs and establish a representative sequence database. The representative sequences were classified and aligned using the SILVA database in Parallel‐Meta3 software (v3.5.2)^46^. The vegan, picante, doBy, ggalt, and ggplot2 packages in the R program were used to perform rarefaction curve and alpha diversity analyses^47^, ^48^. For each sample, 8000 reads were included for the calculation of rarefaction with intervals of 30 and permutations of 5 and PCoA using the Jaccard method based on PAST (v4.10)^49^ was used to assess the dissimilarities between the gill and the adjacent seawater microbial communities.

### FISH and TEM

To determine the location and distribution of the gill symbiont discovered in the present study, we designed FISH probes based on its 16S rRNA gene sequence. The uniqueness and specificity of the probe sequence were checked by aligning the 16S rRNA gene sequence with sequences in the NCBI nucleotide database. The alignments between the 16S rRNA genes of the symbionts and their five best hits with the highest sequence similarities (no more than 89%) were manually checked (Figure S9). The aligned region showing the greatest difference was then manually selected (Figure S9). The specificity of the probe sequence was further confirmed by searching against the NCBI nucleotide database. After that, 30‐bp oligonucleotide probes with 100% matches to the regions were synthesized and labeled with the fluorescent dye Cy5 (Table S1).

Preparatory to the FISH analysis, fresh gill tissues from the scallop *A. i. irradians* were fixed in a 4% formaldehyde‐saltwater solution for 12 h and dehydrated in an increasing methanol gradient (25%, 50%, and 75%, 30 min for each step) and embedded in paraffin. Five micrometers of section were cut using a semiautomatic microtome (Leica Biosystems RM2245). After removal of the wax using xylene and ethanol, the sections were rehydrated in a decreasing ethanol gradient (100%, 95%, 80%, and 70%, 15 min for each step), and then hybridized with 5 μg/ml probes in hybridization buffer (0.9 M NaCl, 0.02 M Tris‐HCl, 0.01% sodium dodecyl sulfate, and 20% formamide) at 46°C for 90 min. The slides were then washed with washing buffer (0.1 M NaCl, 0.02 M Tris‐HCl, 0.01% sodium dodecyl sulfate, and 5 mM ethylenediaminetetraacetic acid) at 48°C for 15 min. After washed twice in PBST buffer (Tween‐20: PBS = 1:1000), DAPI was added to the slides and allowed to incubate at room temperature for 5 min. Images were acquired using a ZEISS LSM 900 (Airyscan 2) confocal laser scanning microscope.

To fix the gill samples for TEM, the scallop gill tissue was dehydrated in an acetone gradient and then transferred to Epon resin (Sigma‐Aldrich) for embedding. An ultramicrotome (Reichert Ultracut S; Leica) was used to slice ultrathin (70 nm) sections that were then stained in 2% aqueous uranyl acetate with lead stain solution (0.3% lead acetate and 0.3% lead nitrate; Sigma‐Aldrich). The subcellular localization of symbionts was confirmed using a JEM‐1200EX TEM (Jeol) at an acceleration voltage of 120 kV.

### Metagenomic sequencing and genome binning

To obtain the genomic information on the gill symbionts, Illumina Novaseq. 6000 (Novogene) sequencing and SQK‐LSK Nanopore sequencing (Oxford Nanopore Technologies) were performed on total DNA isolated from gill tissue, to generate 150‐bp end‐paired reads and long reads, respectively (Table S3). Quality control of sequence reads obtained by Illumina sequencing was performed on a local server using the software NGS QC Toolkit (v2.0)^50^. The Illumina reads with adapters, low‐quality reads (assigned by a quality score <20 and the read length >30%), or unpaired high‐quality reads were removed based on our previous study^51^, ^52^. Real‐time, high‐accuracy base calling (quality cutoff, Q7) of the Nanopore reads was performed using Guppy (v4.0.11) (https://github.com/nanoporetech/pyguppyclient), and the reads after base‐calling were analyzed using NanoPlot (v1.18.2)^53^. The clean Illumina and Nanopore reads were cross‐assembled using OPERA‐MS (v0.9.0)^54^, and the quality of the assembled long contigs was evaluated using QUAST (v4.6.3)^55^. The information on the assembled contigs is listed in Table S4. Genome bins were preliminarily extracted from contigs with lengths greater than 10 000 bp using MaxBin (v2.2.6)^56^, MetaBAT (v2.15.3)^57^, and Concoct (v1.1.0)^58^. The completeness and contamination of metagenome‐assembled genomes (MAGs) were evaluated using CheckM (v1.1.2)^59^. MAGs with contamination less than 5% and completeness over 80% were reassembled following three steps: (1) Illumina and Nanopore reads aligned to the MAG using BLATN (*E*‐value < 1e−7 and similarity >99%) were extracted; (2) Spades software (v3.15.5)^60^ was used to reassemble the Illumina and Nanopore reads in a mixed‐assembly mode; (3) the reassembled MAGs were further qualified using CheckM. The information on the MAGs is shown in Table S4. GTDB‐Tk (v0.3.2)^61^ was used for the taxonomic classification of the MAGs.

### Functional genomic analyses

To investigate the potential function of the scallop symbiont, ORFs were predicted by Prodigal (v2.6.3)^62^ in the single genome mode and annotated by BLASTP against the KEGG database^63^ with *E*‐value < 1e−7. Microbial metabolic pathways were reconstructed and viewed using the online software KEGG Mapper (https://www.genome.jp/kegg/mapper.html). To further explore the function and novelty of the scallop symbiont, sequences of bivalve symbionts belonging to *Gammaproteobacteria* were downloaded as references. Information on all the reference genomes is shown in Table S4, including the GenBank accession numbers, the genome sizes (Mb), and the GC contents. The completeness and GC contents of these reference genomes were evaluated using CheckM (v1.1.2). The ORFs were predicted using Prodigal software (v.2.6.3) and further annotated by searching against the KEGG database on a local server. For phylogenetic analysis, the 16S rRNA genes from all the reference genomes were predicted using Barrnap v0.9 (https://github.com/tseemann/barrnap) and then aligned in the ClustalW mode using MEGA software (v7.0)^64^. The maximum‐likelihood phylogenetic tree, including the scallop symbiont and other symbionts, was then constructed with 1000 replicates. The tree was drawn to scale, and the number of substitutions per site indicated the measured branch length.

### RT‐PCR analysis and metatranscriptomics

To determine the in situ expression of DMSP metabolic genes (*dddP*, *dmdA*, and *dmdB*) in the gill symbiont, total RNA was extracted from the scallop gill tissue using the acidic guanidinium thiocyanate‐phenol‐chloroform method^65^. RNA concentrations and quality were detected by 2% agarose gel electrophoresis and spectrophotometry using a NanoDrop instrument (Wilmington). To avoid genomic DNA contamination, a system containing 2.0 μl of gDNA Clean Reaction (Thermo Fisher Scientific) mix buffer, 1.0 μl of total RNA, and 7.0 μl of RNase‐free ddH_2_O was prepared and the reaction was conducted at 42°C for 2 min. For cDNA synthesis, a system containing 4 μl of 5× Evo M‐MLV RT Reaction (Thermo Fisher Scientific) mix buffer, 6.0 μl of RNase‐free ddH_2_O, and 10 μl of pure RNA was prepared and treated at 37°C for 15 min, 85°C for 5 s, and 4°C for 10 min. The quality of the cDNA products was examined by 2% agarose gel electrophoresis. Specific primers (Table S1) of the target genes were designed for RT‐PCR, while the synthesized cDNA was used as the template. The RT‐PCR reactions were carried out in a system containing 5 μl of 2× Phusion Master Mix (NEB), 0.5 μl of F/R primers, 0.5 μl of cDNA template, and 3.5 μl of ddH_2_O, at 98°C for 30 s, 26 cycles of amplification (98°C for 10 s, 50°C for 30 s, and 72°C for 15 s), and 72°C for 2 min. The products were evaluated on 2% agarose gel electrophoresis.

To further measure the gene or transcript expression levels of genes associated with DMSP metabolism (*dddP, dmdA*, and *dmdB*) in the gill symbiont, the above total RNA of scallop gill tissues was subjected to metatranscriptomic sequencing and analyses. Briefly, the total RNA was combined with biotin‐labeled oligonucleotides corresponding to rRNA or other noncoding RNAs, and the mRNA was selectively maintained and converted to cDNA for library creation. The cDNA libraries were sequenced on the Novaseq. 6000 (Novogene) to generate 20 Gb of data. Clean reads were obtained using the NGS QC Toolkit and then were mapped to the symbiont ORFs using Diamond BLASTn (*E*‐value < 1e−7 and similarity >95%). The TPM value of each gene was calculated by dividing the mapped read counts by the gene lengths in kilobases.

### Heterologous expression and phylogenetic analysis of the *dddP* gene

To verify the enzymatic activity of the *dddP* gene from the gill symbiont, we followed the steps described in a previous study^66^. Briefly, the *dddP* gene in the scallop symbiont was cloned into the expression plasmid *p*ET28a (+) with restriction enzymes NcoI and XhoI and then transferred to *E. coli* BL21(DE3). The *dddP*‐recombinant BL21 strain was cultured in the Luria–Bertani complete medium with kanamycin (100 µg/ml) at 37°C. Recombinant gene expression was initiated by the addition of 0.5 mM isopropyl‐d‐1 thiogalactopyranoside (IPTG) when the growth of recombinant *E. coli* reached OD_600_ = 0.4. The culture was then shaken overnight at 4°C. The control group without IPTG induction was cultured under the same conditions. The bacterial cells were harvested by centrifugation at 5000 rpm for 10 min at 4°C and washed twice with ice‐cold PBS. Expression of the *dddP* gene was confirmed using analytical SDS‐PAGE. The culture was washed twice with M9 medium (Na_2_HPO_4_ **·**2H_2_O 7.52 g/l, KH_2_PO_4_ 3 g/l, NaCl 0.5 g/l, NH_4_Cl 0.5 g/l, sterile ddH_2_O to make 1 l)^67^. Bacterial cells were resuspended in the M9 medium and incubated with 500 nM DMSP for 2 h at 37°C in a shaker. DMS was quantified using a gas chromatography system equipped with a flame photometric detector (GC2014; Shimadzu).

To understand the evolutionary relationship between the *dddP* gene of the scallop gill symbiont and previously‐reported *dddP* genes, we constructed a phylogenetic tree of DddP proteins using MEGA (v7.0). As references, DddP protein sequences (*n* = 50) belonging to different phyla were downloaded from the UniProt database and used. Several of the 50 DddP proteins were predicted to be able to cleave DMSP. For example, *R. nubinhibens* DddP (A3SK19) catabolizes DMSP to DMS, and site‐directed mutations (specifically D295A, D297A, D307A, H371A, E406A, and E421) of DddP entirely abolish this activity^68^. Amino acids were aligned using the ClustalW method, and positions containing gaps and missing data were eliminated. The tree was built in the maximum‐likelihood mode, and bootstrap values were calculated based on 1000 replicates. The tree was drawn to scale, and the number of substitutions per site indicated the measured branch length.

## AUTHOR CONTRIBUTIONS

**Yi Shu:** Data curation (Lead); Investigation (Supporting); Visualization (Lead); Writing—original draft (Supporting). **Yongming Wang:** Data curation (Supporting); Investigation (Lead); Visualization (Supporting); Writing—original draft (Supporting); Writing—review & editing (Supporting). **Zhongcheng Wei:** Data curation (Supporting). **Ning Gao:** Data curation (Supporting). **Shuyan Wang:** Data curation (Supporting). **Chun‐Yang Li:** Data curation (Supporting). **Qiang Xing:** Investigation (Supporting). **Xiaoli Hu:** Conceptualization (Supporting). **Xiaohua Zhang:** Writing—review & editing (Supporting). **Yu‐Zhong Zhong:** Conceptualization (Supporting). **Weipeng Zhang:** Conceptualization (Supporting); Writing—original draft (Supporting); Writing—review & editing (Supporting). **Zhenmin Bao:** Conceptualization (Supporting); Funding acquisition (Lead); **Wei Ding:** Conceptualization (Lead); Funding acquisition (Supporting); Writing—original draft (Lead); Writing—review & editing (Lead).

## ETHICS STATEMENT

All applicable international, national, and/or institutional guidelines for the care and use of animals were followed.

## CONFLICT OF INTERESTS

The authors declare no conflict of interests.

## Supporting information

Supporting information.

Supporting information.

## Data Availability

Raw data of the 16S rRNA sequencing and metatranscriptome have been deposited in the NCBI SRA database under the BioProject number PRJNA880434 and SRA accession number SRR23580857, respectively. The genome sequence of this symbiont has been deposited in the NCBI GenBank database under the accession number JAPKNN000000000. More detailed information is given in Table S4.

## References

[1] Kiene RP , Linn LJ , Bruton JA . New and important roles for DMSP in marine microbial communities. J Sea Res. 2000;43:209–24.

[2] Brock NL , Citron CA , Zell C , Berger M , Wagner‐Döbler I , Petersen J , et al. Isotopically labeled sulfur compounds and synthetic selenium and tellurium analogues to study sulfur metabolism in marine bacteria. Beilstein J Org Chem. 2013;9:942–50. 23766810 10.3762/bjoc.9.108 PMC3678758

[3] Cirri E , Pohnert G . Algae‐bacteria interactions that balance the planktonic microbiome. New Phytol. 2019;223:100–6. 30825329 10.1111/nph.15765

[4] Zinke L . Cool gas in warm summers. Nat Clim Change. 2019;9:434.

[5] Dey M . Enzymology of microbial dimethylsulfoniopropionate catabolism. Adv Protein Chem Struct Biol. 2017;109:195–222. 28683918 10.1016/bs.apcsb.2017.05.001

[6] Thume K , Gebser B , Chen L , Meyer N , Kieber DJ , Pohnert G . The metabolite dimethylsulfoxonium propionate extends the marine organosulfur cycle. Nature. 2018;563:412–5. 30429546 10.1038/s41586-018-0675-0

[7] Zhang XH , Liu J , Liu J , Yang G , Xue CX , Curson ARJ , et al. Biogenic production of DMSP and its degradation to DMS–their roles in the global sulfur cycle. Sci China Life Sci. 2019;62:1296–319. 31231779 10.1007/s11427-018-9524-y

[8] Howard EC , Henriksen JR , Buchan A , Reisch CR , Bürgmann H , Welsh R , et al. Bacterial taxa that limit sulfur flux from the ocean. Science. 2006;314:649–52. 17068264 10.1126/science.1130657

[9] Curson ARJ , Sullivan MJ , Todd JD , Johnston AWB . DddY, a periplasmic dimethylsulfoniopropionate lyase found in taxonomically diverse species of *Proteobacteria* . ISME J. 2011;5:1191–200. 21248856 10.1038/ismej.2010.203 PMC3146280

[10] Li CY , Wang XJ , Chen XL , Sheng Q , Zhang S , Wang P , et al. A novel ATP dependent dimethylsulfoniopropionate lyase in bacteria that releases dimethyl sulfide and acryloyl‐CoA. eLife. 2021;10:e64045. 33970104 10.7554/eLife.64045 PMC8163506

[11] Todd JD , Curson ARJ , Dupont CL , Nicholson P , Johnston AWB . The *dddP* gene, encoding a novel enzyme that converts dimethylsulfoniopropionate into dimethyl sulfide, is widespread in ocean metagenomes and marine bacteria and also occurs in some *Ascomycete* fungi. Environ Microbiol. 2009;11:1376–85. 19220400 10.1111/j.1462-2920.2009.01864.x

[12] Todd JD , Curson ARJ , Nikolaidou‐Katsaraidou N , Brearley CA , Watmough NJ , Chan Y , et al. Molecular dissection of bacterial acrylate catabolism—unexpected links with dimethylsulfoniopropionate catabolism and dimethyl sulfide production. Environ Microbiol. 2010;12:327–43. 19807777 10.1111/j.1462-2920.2009.02071.x

[13] Curson ARJ , Williams BT , Pinchbeck BJ , Sims LP , Martínez AB , Rivera PPL , et al. DSYB catalyses the key step of dimethylsulfoniopropionate biosynthesis in many phytoplankton. Nat Microbiol. 2018;3:430–9. 29483657 10.1038/s41564-018-0119-5

[14] Zheng Y , Wang J , Zhou S , Zhang Y , Liu J , Xue CX , et al. Bacteria are important dimethylsulfoniopropionate producers in marine aphotic and high‐pressure environments. Nat Commun. 2020;11:4658. 32938931 10.1038/s41467-020-18434-4 PMC7494906

[15] Teng ZJ , Qin QL , Zhang W , Li J , Fu HH , Wang P , et al. Biogeographic traits of dimethyl sulfide and dimethylsulfoniopropionate cycling in polar oceans. Microbiome. 2021;9:207. 34654476 10.1186/s40168-021-01153-3 PMC8520302

[16] Raina JB , Tapiolas D , Willis BL , Bourne DG . Coral‐associated bacteria and their role in the biogeochemical cycling of sulfur. Appl Environ Microbiol. 2009;75:3492–501. 19346350 10.1128/AEM.02567-08 PMC2687302

[17] Wang H , Liu J , Li Y , Zhu X , Liu Z . Responses to two‐way selection on growth in mass‐spawned F1 progeny of *Argopecten irradians concentricus* (Say). Chin J Oceanol Limnol. 2014;32:349–57.

[18] Wolfe GV , Steinke M . Grazing‐activated production of dimethyl sulfide (DMS) by two clones of *Emiliania huxleyi* . Limnol Oceanogr. 1996;41:1151–60.

[19] Wolfe GV , Steinke M , Kirst GO . Grazing‐activated chemical defence in a unicellular marine alga. Nature. 1997;387:894–7.

[20] Chen K , Shen SD . The influence on the environment and the promotion of aquacultural animal nutrition of DMSP. Trans Oceanol Limnol. 2002;3:36–45.

[21] Dubilier N , Bergin C , Lott C . Symbiotic diversity in marine animals: the art of harnessing chemosynthesis. Nat Rev Microbiol. 2008;6:725–40. 18794911 10.1038/nrmicro1992

[22] Wentrup C , Wendeberg A , Schimak M , Borowski C , Dubilier N . Forever competent: deep‐sea bivalves are colonized by their chemosynthetic symbionts throughout their lifetime. Environ Microbiol. 2014;16:3699–713. 25142549 10.1111/1462-2920.12597

[23] Decker C , Olu K , Cunha RL , Arnaud‐Haond S . Phylogeny and diversification patterns among *Vesicomyid bivalves* . PLoS One. 2012;7:e33359. 22511920 10.1371/journal.pone.0033359 PMC3325225

[24] Johnson SB , Krylova EM , Audzijonyte A , Sahling H , Vrijenhoek RC . Phylogeny and origins of chemosynthetic *Vesicomyid* clams. System Biodivers. 2017;15:346–60.

[25] Ip JCH , Xu T , Sun J , Li R , Chen C , Lan Y , et al. Host‐endosymbiont genome integration in a deep‐sea chemosymbiotic clam. Mol Biol Evol. 2021;38:502–18. 32956455 10.1093/molbev/msaa241 PMC7826175

[26] Lim SJ , Davis BG , Gill DE , Walton J , Nachman E , Engel AS , et al. Taxonomic and functional heterogeneity of the gill microbiome in a symbiotic coastal mangrove lucinid species. ISME J. 2019;13:902–20. 30518817 10.1038/s41396-018-0318-3 PMC6461927

[27] Wilson ACC , Duncan RP . Signatures of host/symbiont genome coevolution in insect nutritional endosymbioses. Proc Natl Acad Sci USA. 2015;112:10255–61. 26039986 10.1073/pnas.1423305112 PMC4547219

[28] Liu X , Li C , Chen M , Liu B , Yan X , Ning J , et al. Draft genomes of two Atlantic bay scallop subspecies *Argopecten irradians irradians* and *A. i. concentricus* . Sci Data. 2020;7:99. 32251283 10.1038/s41597-020-0441-7 PMC7090048

[29] Curson ARJ , Fowler EK , Dickens S , Johnston AWB , Todd JD . Multiple DMSP lyases in the γ‐proteobacterium *Oceanimonas doudoroffii* . Biogeochemistry. 2012;110:109–19.

[30] Roeselers G , Newton ILG . On the evolutionary ecology of symbioses between chemosynthetic bacteria and bivalves. Appl Microbiol Biotechnol. 2012;94:1–10. 22354364 10.1007/s00253-011-3819-9 PMC3304057

[31] Petersen JM , Zielinski FU , Pape T , Seifert R , Moraru C , Amann R , et al. Hydrogen is an energy source for hydrothermal vent symbioses. Nature. 2011;476:176–80. 21833083 10.1038/nature10325

[32] Duperron S , Gaudron SM , Rodrigues CF , Cunha MR , Decker C , Olu K . An overview of chemosynthetic symbioses in bivalves from the North Atlantic and Mediterranean Sea. Biogeosciences. 2013;10:3241–67.

[33] Brissac T , Rodrigues CF , Gros O , Duperron S . Characterization of bacterial symbioses in Myrtea sp. (Bivalvia: Lucinidae) and Thyasira sp. (Bivalvia: Thyasiridae) from a cold seep in the Eastern Mediterranean: identification of two bivalves and their bacterial symbionts. Mar Ecol. 2011;32:198–210.

[34] Rodrigues CF , Duperron S . Distinct symbiont lineages in three thyasirid species (Bivalvia: *Thyasiridae*) from the eastern Atlantic and Mediterranean Sea. Naturwissenschaften. 2011;98:281–7. 21336695 10.1007/s00114-011-0766-3

[35] Oliver KM , Degnan PH , Burke GR , Moran NA . Facultative symbionts in aphids and the horizontal transfer of ecologically important traits. Annu Rev Entomol. 2010;55:247–66. 19728837 10.1146/annurev-ento-112408-085305

[36] Stewart FJ , Cavanaugh CM . Bacterial endosymbioses in *Solemya* (Mollusca: Bivalvia)—model systems for studies of symbiont‐host adaptation. Antonie Van Leeuwenhoek. 2006;90:343–60. 17028934 10.1007/s10482-006-9086-6

[37] Petersen JM , Kemper A , Gruber‐Vodicka H , Cardini U , van der Geest M , Kleiner M , et al. Chemosynthetic symbionts of marine invertebrate animals are capable of nitrogen fixation. Nat Microbiol. 2016;2:16195. 27775707 10.1038/nmicrobiol.2016.195 PMC6872982

[38] Seah BKB , Antony CP , Huettel B , Zarzycki J , Schada von Borzyskowski L , Erb TJ , et al. Sulfur‐oxidizing symbionts without canonical genes for autotrophic CO_2_ fixation. mBio. 2019;25:e01112‐19. 10.1128/mBio.01112-19 PMC6593406 31239380

[39] Bullock HA , Luo H , Whitman WB . Evolution of dimethylsulfoniopropionate metabolism in marine phytoplankton and bacteria. Front Microbiol. 2017;8:637. 28469605 10.3389/fmicb.2017.00637 PMC5395565

[40] Soen Y . Environmental disruption of host‐microbe co‐adaptation as a potential driving force in evolution. Front Genet. 2014;5:168. 24999350 10.3389/fgene.2014.00168 PMC4064665

[41] Wang S , Su X , Cui H , Wang M , Hu X , Ding W , et al. Microbial richness of marine biofilms revealed by sequencing full‐length 16S rRNA genes. Genes. 2022;13:1050. 35741812 10.3390/genes13061050 PMC9223118

[42] Wang H , Wang M , Fan S , Lu J , Lan Y , Li M , et al. Culture enrichment combined with long‐read sequencing facilitates genomic understanding of hadal sediment microbes. Front Marine Sci. 2021;8:754332.

[43] Martin M . Cutadapt removes adapter sequences from high‐throughput sequencing reads. EMBnet.journal. 2011;17:10–2.

[44] Rognes T , Flouri T , Nichols B , Quince C , Mahé F . VSEARCH: a versatile open source tool for metagenomics. PeerJ. 2016;4:e2584. 27781170 10.7717/peerj.2584 PMC5075697

[45] Edgar RC , Haas BJ , Clemente JC , Quince C , Knight R . UCHIME improves sensitivity and speed of chimera detection. Bioinformatics. 2011;27:2194–200. 21700674 10.1093/bioinformatics/btr381 PMC3150044

[46] Jing G , Sun Z , Wang H , Gong Y , Huang S , Ning K , et al. Parallel‐META 3: comprehensive taxonomical and functional analysis platform for efficient comparison of microbial communities. Sci Rep. 2017;7:40371. 28079128 10.1038/srep40371 PMC5227994

[47] Kembel SW , Cowan PD , Helmus MR , Cornwell WK , Morlon H , Ackerly DD , et al. Picante: R tools for integrating phylogenies and ecology. Bioinformatics. 2010;26:1463–4. 20395285 10.1093/bioinformatics/btq166

[48] Villanueva RAM , Chen ZJ . ggplot2: elegant graphics for data analysis (2nd ed.). Measurement: Interdiscip Res Perspect. 2019;17:160–7.

[49] Hammer‐Muntz O , Harper D , Ryan PD . PAST: paleontological statistics software package for education and data analysis. Palaeontol Electron. 2001;4:1–9.

[50] Patel RK , Jain M . NGS QC toolkit: a toolkit for quality control of next generation sequencing data. PLoS One. 2012;7:e30619. 22312429 10.1371/journal.pone.0030619 PMC3270013

[51] Ding W , Wang S , Qin P , Fan S , Su X , Cai P , et al. Anaerobic thiosulfate oxidation by the Roseobacter group is prevalent in marine biofilms. Nat Commun. 2023;14:2033. 37041201 10.1038/s41467-023-37759-4 PMC10090131

[52] Qin P , Cui H , Li P , Wang S , Fan S , Lu J , et al. Early stage of biofilm assembly on microplastics is structured by substrate size and bacterial motility. iMeta. 2023;e121. 10.1002/imt2.121 PMC10989967 38867926

[53] De Coster W , D'Hert S , Schultz DT , Cruts M , Van Broeckhoven C . NanoPack: visualizing and processing long‐read sequencing data. Bioinformatics. 2018;34:2666–9. 29547981 10.1093/bioinformatics/bty149 PMC6061794

[54] Bertrand D , Shaw J , Kalathiyappan M , Ng AHQ , Kumar MS , Li C , et al. Hybrid metagenomic assembly enables high‐resolution analysis of resistance determinants and mobile elements in human microbiomes. Nat Biotechnol. 2019;37:937–44. 31359005 10.1038/s41587-019-0191-2

[55] Mikheenko A , Valin G , Prjibelski A , Saveliev V , Gurevich A . Icarus: visualizer for de novo assembly evaluation. Bioinformatics. 2016;32:3321–3323. 27378299 10.1093/bioinformatics/btw379

[56] Wu YW , Simmons BA , Singer SW . MaxBin 2.0: an automated binning algorithmto recover genomes from multiple metagenomic datasets. Bioinformatics. 2015;32:605–7. 26515820 10.1093/bioinformatics/btv638

[57] Kang DD , Li F , Kirton E , Thomas A , Egan R , An H , et al. MetaBAT 2: an adaptivebinning algorithm for robust and efficient genome reconstruction from meta‐genome assemblies. Peer J. 2019;7:e7359. 31388474 10.7717/peerj.7359 PMC6662567

[58] Alneberg J , Bjarnason BS , de Bruijn I , Schirmer M , Quick J , Ijaz UZ , et al. Binning metagenomic contigs by coverage and composition. Nat Methods. 2014;11:1144–6. 25218180 10.1038/nmeth.3103

[59] Parks DH , Imelfort M , Skennerton CT , Hugenholtz P , Tyson GW . CheckM: assessing the quality of microbial genomes recovered from isolates, single cells, and metagenomes. Genome Res. 2015;25:1043–55. 25977477 10.1101/gr.186072.114 PMC4484387

[60] Antipov D , Korobeynikov A , McLean JS , Pevzner PA . HYBRIDSPADES: an algorithm for hybrid assembly of short and long reads. Bioinformatics. 2016;32:1009–15. 26589280 10.1093/bioinformatics/btv688 PMC4907386

[61] Chaumeil PA , Mussig AJ , Hugenholtz P , Parks DH . GTDB‐Tk: a toolkit to classify genomes with the genome taxonomy database. Bioinformatics. 2019;36:1925–1927. 31730192 10.1093/bioinformatics/btz848 PMC7703759

[62] Hyatt D , Chen GL , Locascio PF , Land ML , Larimer FW , Hauser LJ . Prodigal: prokaryotic gene recognition and translation initiation site identification. BMC Bioinformatics. 2010;11:119. 20211023 10.1186/1471-2105-11-119 PMC2848648

[63] Kanehisa M . KEGG: kyoto encyclopedia of genes and genomes. Nucleic Acids Res. 2000;28:27–30. 10592173 10.1093/nar/28.1.27 PMC102409

[64] Kumar S , Stecher G , Tamura K . MEGA7: molecular evolutionary genetics analysis version 7.0 for bigger datasets. Mol Biol Evol. 2016;33:1870–4. 27004904 10.1093/molbev/msw054 PMC8210823

[65] Chomczynski P , Sacchi N . Single‐step method of RNA isolation by acid guanidinium thiocyanate‐phenol‐chloroform extraction. Anal Biochem. 1987;162:156–9. 2440339 10.1006/abio.1987.9999

[66] Liu J , Liu J , Zhang SH , Liang J , Lin H , Song D , et al. Novel insights into bacterial dimethylsulfoniopropionate catabolism in the East China Sea. Front Microbiol. 2018;9:3206. 30622530 10.3389/fmicb.2018.03206 PMC6309047

[67] Sambrook J , Fritsch EF , Maniatis T . Molecular cloning: a laboratory manual. Cold Spring Harbor: Cold Spring Harbor Laboratory Press; 1989.

[68] Kirkwood M , Le Brun NE , Todd JD , Johnston AWB . The *dddP* gene of *Roseovarius nubinhibens* encodes a novel lyase that cleaves dimethylsulfoniopropionate into acrylate plus dimethyl sulfide. Microbiology. 2010;156:1900–6. 20378650 10.1099/mic.0.038927-0

