# Supplementary material for: A bacterial symbiont in the gill of the marine scallop Argopecten irradians irradians metabolizes dimethylsulfoniopropionate
Source: mLife. 2023 Jun 26;2(2):178–89. doi: 10.1002/mlf2.12072 (PMC10989825; doi:10.1002/mlf2.12072)
Supplement: Supplementary file 1 — Supporting information. [file MLF2-2-178-s001.docx]

**Figure S1** Rarefaction curves of the observed operational taxonomic unit (OTU) and the 16S rRNA gene sequences. The number of observed OTUs was calculated with 5 permutations.

**Figure S2** Alpha diversity of the gill and the seawater microbiota. (A) Chao_1 index. (B) Shannon index. These results were based on the operational taxonomic unit (OTU) table. Error bars represent standard deviations.


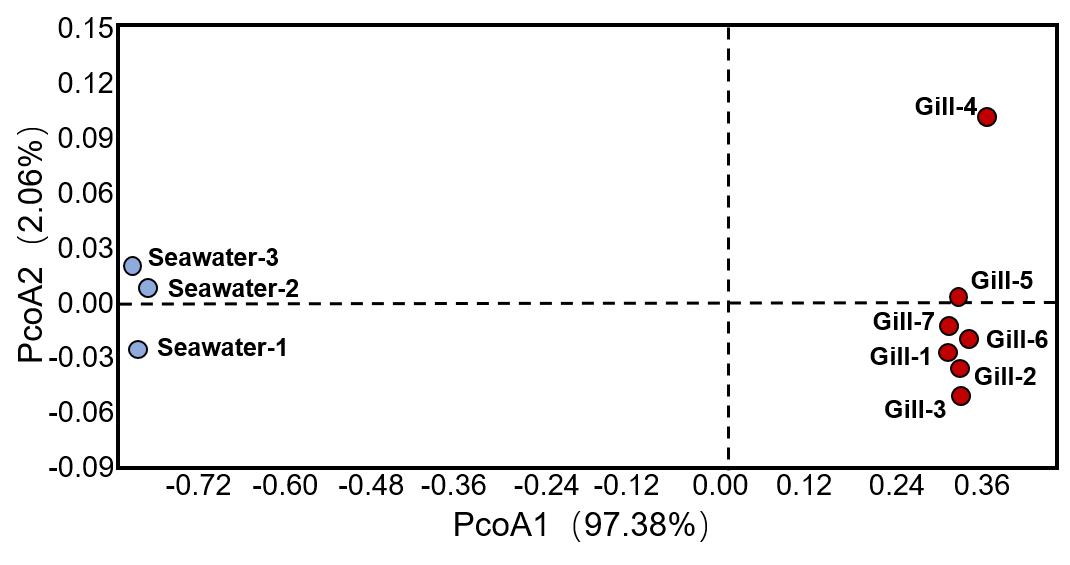


**Figure S3** A plot of principal coordinate analysis (PCoA) to visualize the community structure dissimilarities between the gill and the seawater microbiota. The dissimilarities were derived from the Jaccard distances based on the operational taxonomic unit (OTU) composition.

**Figure S4** Taxonomic community structures of the gill and the seawater microbiota at the phylum level. Proteobacteria were further classified to the class level.

**Figure S5**. Taxonomic community structures of the gill and the seawater microbiota at the genus level. The top 30 genera ranked by the total relative abundance among the 10 samples are shown, while the other genera are summarized as ‘Others’.

**Figure S6** Taxonomic community structure of the gill microbiota at the operational taxonomic unit (OTU) level. Except OTU_322591, all the other OTUs are summarized as ‘Other OTUs’.

**Figure S7** Transmission electron microscopy (TEM) photo of the gill tissues. Gc: gill cells; BM: basilar membrane; N: cell nucleus; MV: microvilli; B: symbiotic bacteria. Scale bar (2 µm) is given at the bottom right.

**Figure S8** The 16S rRNA gene sequence alignment between *Rhodobiaceae* bacterium HWgs001 and *Rhodobacteraceae* bacterium KLH1.

**Figure S9** The 16S rRNA gene sequence alignment between *Rhodobiaceae* bacterium HWgs001 (represented by HWgs001) and other most closely related species in Genbank. Other species: L13 (DQ860071.1); APM10 (JQ347372.1); SHFH652 (FJ203577.1); SHFGG632 (FJ203222.1); Mfav_F16 (GU118597.1); LC1-25 (DQ289899.1); AG2_A11 (KT952653.1); SCS_HX36_102 (HM598213.1); MD2905-B3 (EU386115.1). The sequence region with the greatest difference between HWgs001 and other species is highlighted in a green box. The specific probe sequence of HWgs001 for FISH experiment were chosen from this region.

**Figure S10** Venn analysis of Kyoto Encyclopedia of Genes and Genomes (KEGG)-annotated genes between HWgs001 and seven other symbionts. These strains were identified in the phylogenetic analysis (Figure 2). (**A**) Common and specific KEGG-annotated genes derived from the analyzed genomes. (**B**) Categories of KEGG-annotated genes shared by these genomes. Categories with more than 2 KEGG-annotated genes are shown.

**Figure S11** *In situ* expression of *dddP*, *dmdA,* and *dmdB* genes detected by reverse transcriptase-polymerase chain reaction (RT-PCR). The target band sizes: *ddd*P, 280 bp; *dmd*A, 210 bp; *dmd*B, 210 bp. Control-1: total DNA from the gill was used as PCR template and the product was used as a positive control. Control-2: ddH_2_O was used as PCR template and the product was used as a negative control. Control-3: total DNA from the scallop mantle was used as PCR template and the product was set as a negative control. Gill: cDNA derived from the gill RNA was used as PCR template.

**Figure S12** Metatranscriptomic analysis of the expression of *dddP*, *dmdA* and *dmdB* genes in the gill symbiont. TPM, transcripts per million.

**Figure S13** Phylogenetic analysis for the HWgs001 DddP protein. Reference DddP protein sequences were downloaded from the NCBI protein database. The phylogenetic tree was built in the Maximum Likelihood mode, and the bootstrap values were calculated based on 1,000 replicates. The tree was drawn to scale, and the number of substitutions per site indicated the measured branch length. The DddP proteins with identified DMSP cleavage activity are labelled by triangles.

**Figure S14** Active amino acid sites in DddP proteins. After mutation of the D295, D297, D307, H371, E406, and H421 amino acids (surrounded by black boxes in the plot) into alanine, the DddP protein from *Roseovarius nubinhibens* (A3SK19) lost its function to generate dimethylsulfide from Dimethylsulfoniopropionate, and thus these amino acids were defined as the active sites [11]. Alignment of DddP protein sequences from *R. nubinhibens* (A3SK19), *Symbiodinium* sp. KB8 (A0A812WHH4), HWgs001, *Paraburkholderia ultramafica* (A0A6S7BBE5), and *Oceanimonas doudoroffii* (G5CZE7) showed that those active sites are rather conserved.

**Figure S15** Sodium dodecyl sulfate-polyacrylamide gel electrophoresis (SDS-PAGE) to show the successful heterologous expression of the HWgs001 *dddP* gene in *Escherichia coli* BL21. The expression was induced by adding isopropyl *β*-d-1-thiogalactopyranoside (IPTG). According to the image of SDS-PAGE, the molecular weight of DddP was approximately 55 kDa.

**Table S1 Primer and probe sequences in this study.**

| Primer name | Forward (5'-3') | Reverse (5'-3') |
| --- | --- | --- |
| 27F/1492R | AGAGTTTGATCMTGGCTCAG | GGTTACCTTGTTACGACTT |
| *dddP* F/R | CCGTCAATATCGTCTAAAAAG | GCTCTTTGTTCTATTTTATCG |
| *dmdA* F/R | CCAAACACTTCCCCTTAACG | GGTATCAGACAAAAGATCAC |
| *dmdB* F/R | TGGTTCACAACAGATCATTTCAC | GTATGGCAAACCATGTCACTTC |
| FISH probe | CGATAGCCCGATAACTGCCAAG | |

**Table S2 Information on 16S rRNA gene amplicons.**

| Sample Name | Data size(bp) | Total number of  clean reads | Average of read length(bp) | Maximum of read length (bp) | Minimum of read length (bp) |
| --- | --- | --- | --- | --- | --- |
| Gill-1 | 19,451,376 | 13,581 | 1,432 | 1,508 | 1,382 |
| Gill-2 | 16,175,472 | 11,367 | 1,423 | 1,504 | 1,377 |
| Gill-3 | 17,391,154 | 12,160 | 1,430 | 1,514 | 1,376 |
| Gill-4 | 21,598,996 | 15,104 | 1,430 | 1,515 | 1,373 |
| Gill-5 | 18,630,150 | 13,083 | 1,423 | 1,512 | 1,382 |
| Gill-6 | 14,515,980 | 10,190 | 1,424 | 1,515 | 1,308 |
| Gill-7 | 14,773,924 | 10,369 | 1,424 | 1,515 | 1,376 |
| Seawater-1 | 221,973,211 | 154,670 | 1,435 | 1,521 | 1,302 |
| Seawater-2 | 187,093,786 | 130,362 | 1,435 | 1,518 | 1,339 |
| Seawater-3 | 181,224,298 | 126,329 | 1,435 | 1,519 | 1,303 |

**Table S3 Information on the metagenomic and** **metatranscriptomic datasets.** The Illumina

metagenomic reads and the Nanopore metagenomic reads were assembled together.

|  | Illumina  metagenome | Oxford Nanopore  metagenome | Illumina  metatranscriptome |
| --- | --- | --- | --- |
| Library insert size (bp) | 350 | 10,000 | 350 |
| Clean data size (Gb) | 61.99 | 25.62 | 24.51 |
| GC (%) | 36.41 | 35.68 | 39.52 |
| N50 of read length (bp) | 150 | 14,138 | 150 |
| Maximum of read length (bp) | 150 | 143,127 | 150 |
| Minimum of read length (bp) | 150 | 52 | 150 |
| Total length (bp) | 1,227,803,806 | | NA |
| Number of contigs | 1,264,685 | | NA |
| Number of contigs longer than 10,000 bp | 1,4623 | | NA |
| Number of contigs longer than 25,000 bp | 7,299 | | NA |
| Number of contigs longer than 50,000 bp | 3,992 | | NA |
| Longest contig (bp) | 1,098,722 | | NA |
| \| N50 (bp) \|  \| \| --- \| --- \| | 28,885 | | NA |
| \| N75 (bp) \|  \|  \| \| --- \| --- \| --- \| | 452 | | NA |
| \| GC (%) \|  \| \| --- \| --- \| | 35.36 | | NA |
